# Multi-Scale Feature Pyramid Network: A Heavily Occluded Pedestrian Detection Network Based on ResNet

**DOI:** 10.3390/s21051820

**Published:** 2021-03-05

**Authors:** Xiaotao Shao, Qing Wang, Wei Yang, Yun Chen, Yi Xie, Yan Shen, Zhongli Wang

**Affiliations:** 1 School of Electronic and Information Engineering, Beijing Jiaotong University, Beijing 100044, China; xtshao@bjtu.edu.cn (X.S.); 19125054@bjtu.edu.cn (Q.W.); wyang@bjtu.edu.cn (W.Y.); zlwang@bjtu.edu.cn (Z.W.); 2 Shanghai Aerospace Control Technology Institute, Shanghai 201109, China; chenyun3305@gmail.com; 3 Beijing Xinghang Mechanical-Electrical Equipment Co., Ltd., Beijing 100074, China; yixie.429@gmail.com

**Keywords:** pedestrian detection, heavy occlusion handling, feature enhancement, MFPN

## Abstract

The existing pedestrian detection algorithms cannot effectively extract features of heavily occluded targets which results in lower detection accuracy. To solve the heavy occlusion in crowds, we propose a multi-scale feature pyramid network based on ResNet (MFPN) to enhance the features of occluded targets and improve the detection accuracy. MFPN includes two modules, namely double feature pyramid network (FPN) integrated with ResNet (DFR) and repulsion loss of minimum (RLM). We propose the double FPN which improves the architecture to further enhance the semantic information and contours of occluded pedestrians, and provide a new way for feature extraction of occluded targets. The features extracted by our network can be more separated and clearer, especially those heavily occluded pedestrians. Repulsion loss is introduced to improve the loss function which can keep predicted boxes away from the ground truths of the unrelated targets. Experiments carried out on the public CrowdHuman dataset, we obtain 90.96% AP which yields the best performance, 5.16% AP gains compared to the FPN-ResNet50 baseline. Compared with the state-of-the-art works, the performance of the pedestrian detection system has been boosted with our method.

## 1. Introduction

Pedestrian detection has been a hot topic in the field of computer vision, widely applied in recent years in both research and industry applications such as automatic driving, video surveillance, human-machine interaction, etc. Many scholars have endeavored to solve the problem in these application fields with great results [1,2]. The problem of pedestrian detection can be simplified as a binary classification between foregrounds (pedestrians) and backgrounds. However, pedestrians are usually exposed to complex environments. For instance, images captured by intelligent vehicles driving in urban scenarios have intricate backgrounds, which will bring about difficulties for pedestrian detection. Meanwhile, pedestrian targets in natural environments are inevitably occluded by each other. Occlusion problems can be classified as inter-class occlusion and intra-class occlusion. The former refers to targets being occluded by objects of different classes, the latter occurs when targets of the same kind are occluded by each other. Both of them remain a huge challenge for pedestrian detection tasks in practice. Especially in the case of highly crowded scenes, it is difficult for pedestrian detectors to precisely detect targets. For example, the CrowdHuman dataset [3] has an average of 23 pedestrians in each image, with various levels of occlusion. It is difficult for general object detectors to solve this kind of problem.

Feature extraction is the key to pedestrian detection. Most existing feature extraction methods can be summarized as traditional hand-crafted features and convolutional neural network (CNN) features. The hand-crafted feature extraction methods such as HOG [4], Haar [5], LBP [6], etc., have to be artificially distinguished whether they are representative or not. Furthermore, it is generally difficult to design features with robustness in practical and complex backgrounds. The CNN features generally refer to features extracted by CNNs, such as AlexNet [7], VGGNet [8], STINet [9], etc., which can extract more complicated and semantic features and result in higher detection accuracy. Therefore, CNN has a wider range of applications in pedestrian detection. However, the features of heavily occluded pedestrians are too similar to distinguish for the detector. To better distinguish the features, Zhang et al. [10] proposed that pedestrians could be respectively divided into several parts, whose features were then extracted partially for further fusion. However, the method requires a much longer time to extract and fuse features which results in a cumbersome training process.

In this paper, we propose a novel scheme called MFPN for heavily occluded pedestrian detection. The main contributions of this paper are two-fold: (1) We propose a novel feature extraction network called double FPN integrated with ResNet (DFR) to enhance the semantic information and contours of occluded pedestrians, and to simplify the network structure and the parameters. (2) We introduce the concept of repulsion loss of minimum (RLM) to keep predicted boxes away from the ground truths of the other pedestrians, which can also monitor the learning process of predicted boxes. The repulsion loss [11] is introduced based on original loss, which can not only reduce the loss between predicted boxes and ground truths but also repel the predicted boxes from their surrounding ground truths which are not their targets. The proposed MFPN can solve the heavy occlusion issue existing in pedestrian detection. Furthermore, the object detector with our RLM can achieve higher detection accuracy.

The remainder of this paper is organized as follows: In Section 2, the methods of pedestrian detection in crowded scenes are introduced. Section 3 describes our crucial MFPN network. Section 4 introduces our experimental processes and discusses the results in detail. Conclusions are presented in Section 5.

## 2. Related Works

Deep learning has been increasingly utilized in computer vision tasks recently. Especially the emergence of CNN has resulted in a breakthrough in the research on object detection [12,13,14,15] and other computer vision tasks [16]. Object detection methods based on deep learning are classified into two categories: one-stage detection and two-stage detection.

The one-stage detectors [12,17,18,19,20,21,22,23], represented by YOLO and SSD, have broken through the detection speed bottleneck of two-stage detectors. The two-stage detectors [24,25,26,27] represented by R-CNN, Fast R-CNN, and Faster R-CNN and based on region proposal network (RPN) have higher detection accuracy but lower speed compared to the one-stage detectors. As the key of the two-stage algorithms, RPN is utilized to generate anchors of different sizes and scales on feature maps. Then, post-processing methods such as non-maximum suppression (NMS) are used to remove duplicate proposals. Although there are no RPNs in the one-stage algorithm, it uses predefined anchors, which can be regressed on feature maps directly. These detection networks can be applied to specific object detection tasks including pedestrian detection, vehicle detection, face detection, etc.

Although these methods are reported to achieve outstanding performance on some datasets, low detection accuracy or a high number of missed detections will occur in the heavily occluded pedestrian detection task. As mentioned in the previous paragraph, the predictions are likely to be mistakenly suppressed by NMS since pedestrians may heavily overlap with each other. Optimized NMS solutions to the problem have been found such as Soft-NMS [28], Softer-NMS [29], Adaptive-NMS [30], etc. Soft-NMS and Softer-NMS were proposed to reduce missed detection of traditional NMS in heavily occluded object detection. Adaptive NMS was proposed to set the threshold of confidence based on the density of targets and to adapt to different levels of occlusions. However, such heuristic variants of NMS cannot be applied flexibly under different circumstances. Furthermore, they cannot solve the heavy occlusion problem fundamentally.

Some works have addressed the occlusion problem from other different perspectives, such as loss function, part-based anchors, and improved network:

(1) *Loss Function*. Wang et al. [11] proposed repulsion loss to optimize the original loss function, attempting to solve the problem of pedestrian detection in crowded scenes. Zhang et al. [10] proposed aggregation loss, which enabled the bounding boxes to be close to ground truths and locate compactly. Although these loss functions can help improve the detection accuracy to some degree, it is difficult to recall overlapped proposals due to the use of traditional NMS.
(2) *Part-based Anchors.* Zhou et al. [31] presented a method of pedestrian detection: whereby the whole body and visible parts of pedestrians were respectively located by regressing two bounding boxes. Chi et al. [32] proposed Pedhunter which can handle occlusion in pedestrian detection. However, the methods are complicated and take a long time.
(3) *Improved Network.* Pang et al. [33] proposed the mask-guided attention network (MGAN) which emphasized visible parts of pedestrians and adjusted overall features to suppress invisible areas and detect occluded pedestrians. Zhang et al. [10] proposed occlusion-aware R-CNN, which divided pedestrians into several parts and extracted features for further fusion. Wang et al. [34] used compositional convolutional neural networks to detect objects. Wu et al. [35] exploited the local temporal context of pedestrians in videos and proposed a tube feature aggregation network (TFAN) aiming to detect occluded pedestrians. However, these methods are too intricate to implement or not robust enough to heavily occluded pedestrians.

Apart from the methods mentioned above, Cao et al. [36] paid more attention to predicted bounding boxes with worse location precision and extracted more contextual information around objects to detect pedestrians. However, this method was only validated on the CityPersons and Caltech datasets. Therefore, experiments carried out on more crowded datasets such as CrowdHuman cannot necessarily be expected to produce the same results.

## 3. Materials and Methods

In this section, we will elaborate our approach for heavily occluded pedestrian detection at length. Our proposed network MFPN is motivated by the following considerations:

(1) Intra-class occlusion. Intra-class occlusion and inter-class occlusion generally exist in pedestrian detection. For intra-class occlusion, the features of targets in the same class are equally important. Different levels of extracting similar features and expressing these similar features will influence the later detection. For example, there will be more false positives occur if the expression ability of features is low. The feature extraction network should not only extract and fuse high-level semantic features but also retain the contour information.
(2) Loss function related to occluded pedestrians. The loss function also influences the detection accuracy of occluded targets in pedestrian detection systems. To repel predicted boxes from surrounding ground truths of other pedestrians in crowds, we introduce the repulsion loss [11] integrated with our minimum of two losses to separate irrelevant boxes.

We formulate the details of our approach as follows: In Section 3.1, we introduce the whole architecture network based on CNN. Section 3.2 describes our crucial DFR network. Finally, Section 3.3 depicts our novel loss in detail.

### 3.1. Architecture Network

**Figure 1:**
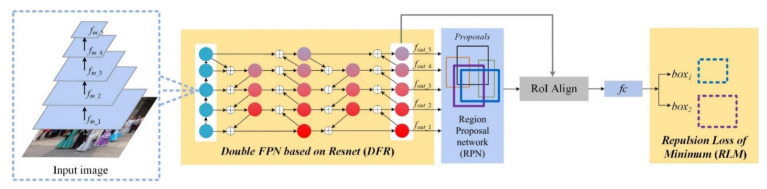
Overall network architecture of MFPN. Feature maps *f_out__*_1_*–f_out__*_5_ are extracted by ResNet50. Two modules with a yellow highlighted background are our DFR and RLM modules. DFR is the feature fusion module to fuse feature maps with different resolutions. We utilize the weighted feature fusion to sum up feature maps distinguishingly, where “⊕” represents the fusion process. The predictions *box*_1_ and *box*_2_ are two predictional boxes from the same proposal. Our new loss function RLM can keep predicted boxes away from other ground truths.

**Figure 2:**
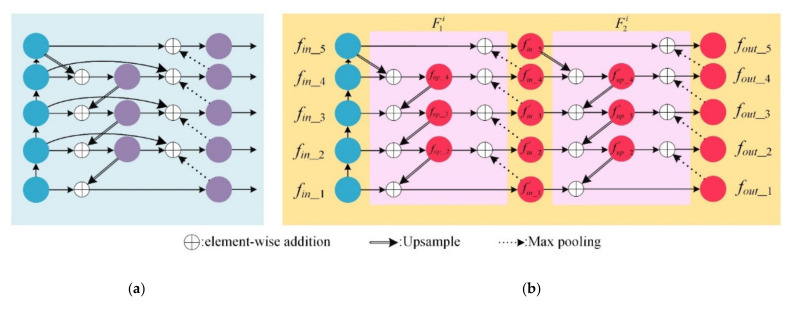
BiFPN and our DFR network. (**a**) BiFPN. The structure of BiFPN is the optimization of FPN and PANet. It utilizes cross-scale connection and weighted feature fusion to extract and fuse features of different resolutions base on the EfficientNet backbone [23]. (**b**) DFR network. We stack two BiFPN layers of bottom-up and top-down structures and then combine the structure with ResNet50, which is widely used in the object detection process to achieve a sound performance of feature extraction and fusion. The blue circles on the left are feature maps extracted by ResNet50, and the remaining on the right is the Double FPN structure we proposed. “⊕” represents the fusion process. The sign of double lines with an arrow “⟹” represents the operation of upsampling which can resize the feature map twice as large as the original. The dashed arrow means the operation of max pooling, which can resize the feature map half of the original.

**Figure 3:**
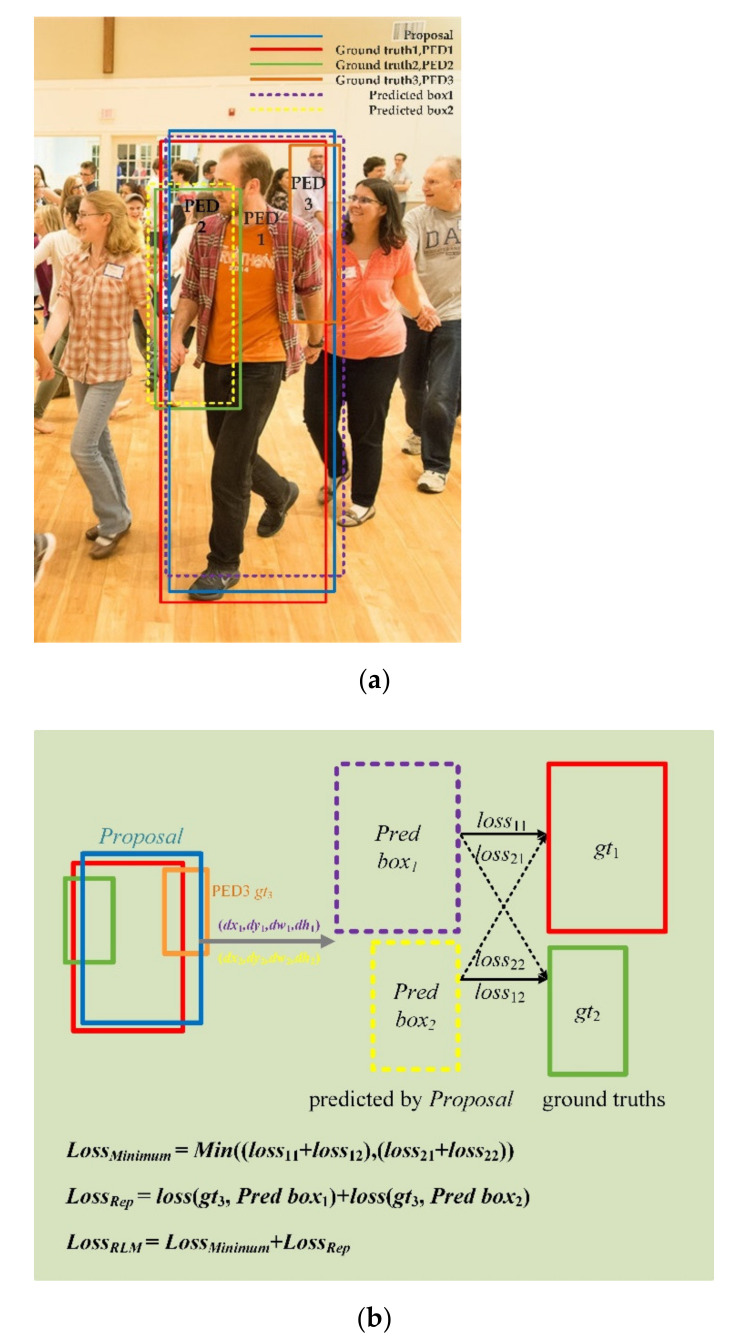
Repulsion loss of minimum. (**a**) Solid boxes(red, green and orange) except for the blue proposal represent ground truths. Dashed boxes (purple and yellow) indicate two predictions by the same blue proposal. (**b**) Dashed (purple and yellow) boxes are predicted boxes by the blue proposal B. Solid boxes, red, green and orange (*gt*_1_, *gt*_2_, *gt*_3_) are the ground truths that have the maximum IoU with the blue proposal. Calculate the loss between the orange box and yellow and purple boxes, resulting in *Loss_Rep_*.

The overview of our MFPN network is shown in Figure 1, which is mainly composed of four parts: DFR, RPN, RoI Align and RLM. In this paper, we use the two-stage detection method and choose ResNet50 as our backbone, followed by our DFR, shown in Figure 2, which is an improved network compared with the traditional FPN. As mentioned in the related works, the RPN of two-stage detection is responsible for generating various anchors on multi-scale feature maps. Some proposals regressed by anchors have been displayed in the RPN module (blue) in Figure 1. The RoI Align module can transform the feature maps of different sizes to the same size combined with the feature maps from *f_out_1_* to *f_out_5_* and information of scales. Then the fully-connected network can predict offsets towards one proposal. The last one is our RLM, composed of *Loss_Minimum_* and *Loss_Rep_*. The loss function diagram will be shown in detail in Figure 3. The *Loss_Rep_* can be applied to the network flexibly.

### 3.2. DFR Network

Effectively extracting and fusing features are of great importance for pedestrian detection tasks. Therefore, feature maps extracted by feature extraction network directly affect the detection accuracy. To achieve the purpose of integrating high-level features with more abstract semantic information and low-level features with more contour information, Lin et al. [37] proposed a pioneering idea: a top-down pathway that can be combined with bottom-up networks to extract and fuse multi-scale features. Following the idea, PANet [38] added an extra bottom-up path on top of FPN. Tan et al. [17] proposed a weighted BiFPN structure that can be scaled to fuse features. The cross-scale connection is proposed in BiFPN to integrate the above network structure, as shown in Figure 2a. Although these works seem to optimize network extraction and fusion and get better accuracy, there are some defects to be overcome: (1) It is still a limit to extracting features of heavily occluded targets for these networks. (2) The overwhelming parameters and computations decrease the efficiency of networks. (3) The networks cannot be employed flexibly.

**Figure 4:**
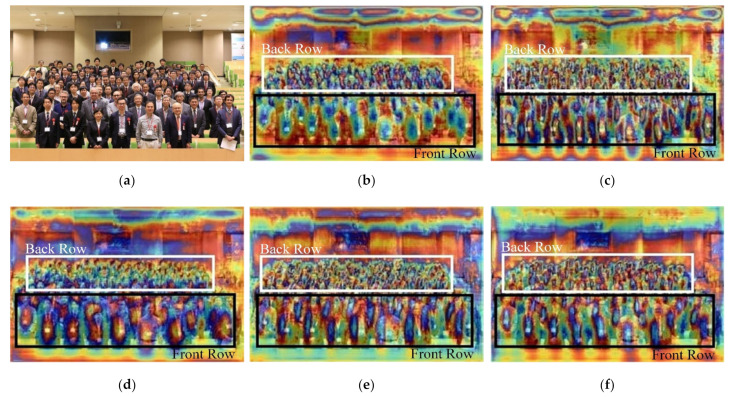
Comparisons of feature maps extracted by different networks. As shown in (**b**), the feature map extracted by ResNet50 and FPN is the worst, with unclear key features and contours. After the combination of ResNet50 and BiFPN (**c**,**d**), the features in the front row are better than (**b**): contours of features in the front row are gradually clearer, and features of key parts can be displayed clearly. (**e**,**f**) are feature maps extracted by our network. The only difference is that (**f**) uses general convolution. It can be seen that the contours and key parts of the features can be clearly represented, especially the features of the targets in the front row. The contours of features extracted by our network in the back row are also gradually separated and clear. Furthermore, we can find that the effect of general convolution on feature extraction network is better than that of separable convolution, comparing (**d**) with (**c**), (**f**) with (**e**). Under the same conditions, separable convolution has fewer parameters than general convolution. Therefore, it is usually used in some lightweight structures. Due to the purpose of improving the performance of the pedestrian detection system, the general convolution is utilized with the factor of parameters ignored. (**a**) Input Image, (**b**) ResNet50 + FPN(GC), (**c**) ResNet50 + BiFPN(SC), (**d**) ResNet50 + BiFPN(GC), (**e**) ResNet50 + Double FPN(SC), (**f**) ResNet50 + Double FPN(GC).

Inspired by previous works, we propose the DFR network, an effective multi-scale feature extraction and fusion network, shown in Figure 2b. The overall DFR is composed of two components, ResNet50 and the double FPN network, which is an improved network compared to ResNet and FPN. We choose ResNet50 as the bottom-up path to extract features because experiments reported in [39] reveal that ResNet50 has low error rates compared with ResNet18 and ResNet34 and requires half the number of parameters compared with ResNet101 and ResNet152. However, ResNet50, ResNet101, and ResNet152 can achieve almost the same accuracy. Our proposed double FPN with ResNet50 (DFR, Figure 2b) makes the following improvements based on the two layers of BiFPN (Figure 2a): First, the connection between the initial input and output of the same level is removed, and then the weight in the corresponding feature fusion should also be removed, which has the advantage of reducing initialization parameters. Second, the separable convolution of the original BiFPN initially introduced in [40], is replaced by a general convolution. Although the network architecture with separable convolution has fewer parameters, experimental results indicate our network with separable convolution cannot surpass the general convolution in all criteria. Meanwhile, in our experiments, the feature maps (Figure 4) extracted by the general-convolution network are clearer than those of separable convolution, which means our network with general convolution can better extract and fuse the features. Therefore, general convolution is better for our network. The modified network structure is shown in Figure 2b.

Suppose the feature map *f_in_i_* extracted by ResNet50 at layer *i* with dimension *h_i_* × *ω_i_* × *c_i_*, *i*∈ ℕ. Let $F_{1,2}^{i}$(pink rectangle background) be the novel function that accomplishes the mapping from *f_in_i_* to *f_out_i_*, denoted as:

(1) $$\begin{array}{ll} F_{1}^{i}=f_{in\_i}\mapsto f_{in\_i}^{\prime },F_{2}^{i}=f_{in\_i}^{\prime }\mapsto f_{out\_i} & \\ where,\;f_{in\_i}={\mathbb{R}}^{h_{i}\times w_{i}\times 2^{i+C_{1}}}, & \\ \;\;\;f_{out\_i}={\mathbb{R}}^{h_{i}\times w_{i}\times 2^{1+C_{1}}}; & \\ and,\;(h_{i},w_{i})=(H,W)/2^{i+C_{2}},i=1,2,3,4,5 \end{array}$$

where the two same operations $F_{1}^{i}$ and $F_{2}^{i}$ are composed of two operations respectively. Parameters *H* and *W* are the height and width of the input image, generally H = 800 and W = 1400 for the CrowdHuman dataset. ${\mathbb{R}}^{h_{i}\times w_{i}\times c_{i}}$ is the dimension of feature maps, where *c_i_in_* = 2*^i^*^+*C*1^, *c_i_out_* = 2^1+*C*1^ are the numbers of channels, and *h_i_* × *ω_i_* is the resolution of feature maps. Here, we let *C*_1_ = 7, *C*_2_ = 1.

For brevity, we only demonstrate $F_{1}^{i}$ in detail, which is made up of two parts. The first one is from *f_in_*_*_i_* (*i* = 1, 2, 3, 4, 5) to *f_up_*_*_i_* (*i* = 2, 3, 4), which can be defined as:

(2) $$\begin{array}{l} f_{up\_i}=F_{Conv}(\frac{\omega_{in\_i}\cdot f_{in\_i}+\omega_{(i+1)i}\cdot F_{Upsample}(f_{up\_i+1})}{\omega_{in\_i}+\omega_{(i+1)i}}),i=2,3\\ f_{up\_4}=F_{Conv}(\frac{\omega_{in\_4}\cdot f_{in\_4}+\omega_{54}\cdot F_{Upsample}(f_{in\_5})}{\omega_{in\_4}+\omega_{54}}) \end{array}$$

where *F_Upsample_* and *F_Conv_* are the operations of upsample and convolution. Notably, the channel of the feature map *f_in_*__5_ is the same as *f_in_*__4_, but the resolution of the former is the quarter of the latter because *f_in_*__5_ is obtained by max pooling *f_in_*__4_. The channels of feature maps except for *f_in_*__2_*−f_in_*__5_ are all 256 for the sake of adding different layers.

The second part is from $f_{up\_i}\;(i=2,3,4)$ to $f_{in\_i}^{\prime }\;(i=1,2,3,4,5)$, which can be defined as:

(3) $$\begin{array}{l} f_{in\_i}^{\prime }=F_{Conv}(\frac{\omega_{up\_i}\cdot f_{up\_i}+\omega_{(i-1)i}\cdot F_{Maxpooling}(f_{in\_i-1}^{\prime })}{\omega_{up\_i}+\omega_{(i-1)i}}),i=2,3,4\\ f_{in\_5}^{\prime }=F_{Conv}(\frac{\omega_{in\_5}\cdot f_{in\_5}+\omega_{45}\cdot F_{Maxpooling}(f_{in\_4}^{\prime })}{\omega_{in\_5}+\omega_{45}}),\\ f_{in\_1}^{\prime }=F_{Conv}(\frac{\omega_{in\_1}\cdot f_{in\_1}+\omega_{21}\cdot F_{Upsample}(f_{up\_2})}{\omega_{in\_1}+\omega_{21}}) \end{array}$$

where *F_Maxpooling_* is the max pooling operation. As shown in Figure 2b, the Equation (1)–(3) only demonstrate the operation $F_{1}^{i}$ reaching out intermediate results of feature maps $f_{in\_1}^{\prime }-f_{in\_5}^{\prime }$, and the final results *f_out_i_* will be obtained through the same operation $F_{2}^{i}$.

Previous works resize feature maps in different layers with a 1 × 1 convolution kernel and sum them up directly, resulting in different layers corresponding to the same importance. However, different layers contribute to the output feature unequally. The weighted feature fusion can learn the importance of feature maps in each layer. Each resized feature map (upsample or max pooling) will be summed up with the next layer’s feature map. The weighted feature fusion has been described in Equations (2) and (3), where *ω* is the weight corresponding to the feature map. Specifically, *ω_in_i_* and *ω*_(*i+*1)*i*_ are on the top-down pathway; *ω_up_j_* and *ω*_(*j−*1)*j*_ are on the bottom-up pathway, which are all the learned weights representing the importance of feature maps for feature fusion. After each feature fusion, *F_conv_* (general convolution) is used to process the feature maps.

### 3.3. Repulsion Loss of Minimum

In this paper, we adopted the paradigm of [41]. That is, for one proposal, two or more bounding boxes can be predicted, which can avoid the situation when bounding boxes are heavily overlapped and only one box survives after the post-processing of NMS. The loss function in pedestrian detection is composed of *regression loss* and *classification loss*. Based on the paradigm, we propose the regression loss termed Repulsion Loss of Minimum (RLM), an improved method of loss function compared to the original in [41], which only uses the first part of our loss function. The classification loss follows the common definition in object detection. In this section, we will detail the RLM loss function to tackle the occlusion problem in pedestrian detection. The repulsion loss of minimum is made up of two components, defined as:

(4) $$Loss_{RLM}=Loss_{Minimum}+Loss_{Rep}$$

As shown in Figure 3a, image, which can not be detected easily and precisely. Let two people, for example PED1 and PED2, be the pedestrians we want to detect. The red and the green boxes are their ground truths, respectively. The blue box is the proposal responsible for locating PED1 and PED2. We can get two sets of offsets towards the proposal further calculate the locations of *Pred box*_1_ and *Pred box*_2_, described as:

(5) $$\left(dx_{i},dy_{i},dw_{i},dh_{i}),i\in (1,2\right)$$

Which ground truth of the pedestrian do the purple *Pred box*_1_ and the yellow *Pred box*_2_ correspond to, respectively? There is the solution that for the two boxes predicted by one proposal, we can calculate a total of two losses corresponding to two pedestrians, respectively, and choose the minimum of the loss. In this case, the pedestrians corresponding to the two boxes are the instances to be predicted. As shown in Figure 3b, the purple *Pred box*_1_ and yellow *Pred box*_2_ are predicted from the same blue proposal. There are two cases of predictions matching specific instances. The first one is that the purple *Pred box*_1_ needs to predict PED1 which the red ground truth (*gt*_1_) represents, and the loss between the two boxes is denoted as $loss_{11}$; the yellow *Pred box*_2_ needs to predict PED2 which the green ground truth (*gt*_2_) represents, and the loss between the two boxes is denoted as $loss_{12}$. Therefore the total loss of the first case is:

(6) $$loss_{1}=loss_{11}+loss_{12}$$

The second one is that the purple *Pred box*_1_ needs to predict PED2 which the green ground truth (*gt*_2_) represents, and the loss between the two boxes is denoted as $loss_{21}$; the yellow *Pred box*_2_ needs to predict PED1 which the red ground truth (*gt*_1_) represents, and the loss between the two boxes is denoted as $loss_{22}$. The total loss of the second case is:

(7) $$loss_{2}=loss_{21}+loss_{22}$$

If *loss*_1_ > *loss*_2_, the second case is selected as the final prediction result, that is:

(8) $$Loss_{Minimum}=Min((loss_{11}+loss_{12}),(loss_{21}+loss_{22}))$$

vice versa. The smaller the loss is, the better the object detector is. That is just where the term “loss of minimum” comes from.

Next is the second *Loss_Rep_* which is firstly proposed in [11]. The proposed *Loss_Rep_* is to keep the two predicted boxes (*Pred box*_1_ and *Pred box*_2_) away from ground truths of other surrounding pedestrians. In this paper, with the dataset CrowdHuman, there are only two classes: pedestrian and background, so the parameter *K*, the number of pedestrians one proposal predicts, should be set as 2. Let P_+_ = {*P*_1_, *P*_2_, …, *P_n_*} represents the positive proposals regressed from anchors. As shown in Figure 3b, the *Loss_Rep_* is defined as:

(9) $$Loss_{Rep}=loss(gt_{3},Predbox_{1})+loss(gt_{3},Predbox2))$$

Generally, for the *n-*th proposal *P_n_*, its repulsion ground truth is defined as the ground truth pedestrian with which it has the largest IoU region except for its designated two targets:

(10) $$G_{Rep}^{P_{n}}=\underset{G\in G\;\backslash \;\{G_{1,2}^{P_{n}}\}}{\arg\max}IoU(G,P_{n})$$

where G = {*G*} is denoted as the set of all ground truths in the image, $G_{1,2}^{P_{n}}=\underset{G\in G}{\arg\max}\;IoU(G,P_{n})$ are the two ground truth pedestrians corresponding to the proposal *P_n_*. The *Loss_Rep_* is between the ground truth $G_{Rep}^{P_{n}}$ and two predicted boxes $B_{1,2}^{P_{n}}$ regressed by *P_n_*, denoted as:

(11) $$Loss_{Rep}=\frac{\sum_{P_{n}\in P_{+}}{\mathrm{Smooth}}_{ln}(IoG(B_{1,2}^{P_{n}},G_{Rep}^{P_{n}}))}{2\left|P_{+}\right|}$$

where:

(12) $$IoG(B,G)=\frac{area(B\cap G)}{area(G)}\in [0,1]$$

and:

(13) $${\mathrm{Smooth}}_{ln}=\left\{\begin{array}{cc} -\ln(1-x) & x\leq \sigma \\ \frac{x-\sigma }{1-\sigma }-\ln(1-\sigma ) & x>\sigma \end{array}\right.$$

Smooth*_ln_* is a smoothed *ln* function, which is continuously differentiable in (0,1). The parameter *σ* is the smooth parameter to adjust the sensitiveness of the repulsion loss to outliers. From Equations (11) and (13), we can find that the more the proposal tends to overlap with a non-target ground truth pedestrian, the larger penalty the *Loss_Rep_* will add to the bounding box regressor. In this way, the *Loss_Rep_* can effectively prevent the predicted bounding boxes from moving to their neighboring pedestrians which are not their targets. Therefore, we expect a higher *Loss_Rep_*, which suggests that the predicted boxes are far from other ground truths, further lead to detect the heavily occluded pedestrians correctly.

## 4. Experiments

In this section, we will compare our method with existing methods in several aspects to verify the effectiveness of our method. The experiment section is organized as follows: (1) We will introduce some basic settings of our experiments: datasets, detailed settings, and experiment platform. (2) We compare the feature maps extracted by our proposed network DFR and previous network, ResNet50 and FPN. (3) The two-stage detector with our method is compared with some other methods on the CrowdHuman and CityPersons datasets.

### 4.1. Datasets

We use the CrowdHuman and CityPersons datasets to evaluate the effectiveness of our method. The CrowdHuman dataset [3] has a large scale, containing 15,000, 4370, and 5000 images for training, validation, and testing, respectively, rich annotations and high diversity. There are about 23 pedestrians in each image with various occlusion scenes. Compared with other datasets, like COCOPerson, Caltech, and CityPersons, the CrowdHuman dataset has more pedestrians in each image, which is a challenge for any pedestrian detector. The CityPersons dataset is widely used in pedestrian detection, which is built on the Cityscapes dataset, a dataset for the task of semantic segmentation in urban street scenes. It includes 2975, 500 and 1525 images for training, validation and testing, respectively. Compared with the CrowdHuman dataset, there are 7 pedestrians in each image. Therefore, experimental results on the two datasets can manifest the effectiveness of our method more persuasively.

### 4.2. Detailed Settings

We use the frequently-used two-stage algorithm of Faster RCNN for the baseline network. The ResNet50 pre-trained on ImageNet is adopted for all the experiments, followed by double FPN. We use RoI Align instead of RoI pooling in this network. For every experiment, we train in total 35 epochs. As for the learning rate, we adopt the method of warming up: every time we train from scratch, the learning rate is gradually and linearly increasing from 0.0001 to 0.001 from iteration 1 to iteration 800 and then remains unchanged. By the 24th epoch, the learning rate is changed to one-tenth of the original, and one percent in the 28th epoch, *i.t.* 0.00001. In our work, due to the multi-size of feature maps, we just need to generate three anchors with different scales such as {1:1, 1:2, 2:1} for both datasets. In the train and test process, K = 2. The sampling ratio of positive to negative proposals for the RoI branch is 1:1 for the CrowdHuman dataset and 1:3 for the CityPersons dataset. All box overlap IoU thresholds are set as 0.5.

### 4.3. Experiment Platform

We implement our network with the PyTorch framework running on a PC equipped with an Intel^®^ Core^TM^ i7-6500U CPU @ 2.50 GHz and an NVIDIA GTX1080Ti GPU. The machine is running Linux Ubuntu 16.04 with NVIDIA CUDA 10.1 and cuDNN 7.0.

### 4.4. Comparison of Feature Maps

In this section, we compare in detail the feature maps extracted by our ResNet50 and double FPN with counterparts extracted by other network structures. For the sake of fairness, an image of the CrowdHuman dataset has been randomly selected for the experiment, and different network structures are used to compare the extracted feature maps with the maximum scale. Table 1 shows different combinations of network structures. Figure 4 shows the feature maps extracted by these networks. We determine the quality of feature maps according to their clarity. For the sake of our explanation, we divide the features of persons into two parts, boxed up by white and black rectangles respectively. The top (bottom) represents the smaller (bigger) targets in the back (front) row. We can find the clarity of contours of these targets are really different in various feature maps. Although it is much easier to extract obscure features of targets in the back row (heavily occluded, white marked), the features extracted by our network can be more separated and clearer, especially those occluded persons in the back row. The contours of those “bigger’’ persons in the front row (light occluded, black marked) are also separated rather than mixed, which can help the detector learn the location of persons.

### 4.5. Ablation Study

In this section, we carry out the ablation experiments on the CrowdHuman dataset. Table 2 shows the experimental results of the proposed approach in previous works, including BiFPN, Separable Convolution, and our double FPN and RLM. The method without “separable” uses general convolution. The baseline network is ResNet50 and FPN [37] which uses NMS for post-processing. The remaining networks in Table 2 all utilize *Set-NMS* [41]. Obviously, our method, which achieves 90.96% AP, 40.24% MR^−2^, 83.12% JI, yields the best performance on the CrowdHuman dataset. Compared to the baseline, our approach achieves 5.16%, 2.66%, and 3.32% improvements in AP, MR^−2^, and JI, respectively. The experimental results indicate that our proposed method without RLM is equipped with good performance in all criteria: 0.58%, 1.68%, and 0.92% improvements even compared with the state–of–the–art method [41]. The improvement indicates that our network can detect more objects accurately although there are heavily occluded targets. At the same time, the improvement of MR^−2^ demonstrates that more false predictions are not introduced. For the separable convolution, the experimental result manifests that the performance of our network with general convolution is all better in three criteria than using separable convolution. Moreover, the results show RLM affects little our network, which only obtains improvements less than 0.5% in all criteria. The performance of our network without RLM decreases by 0.08%, 0.28% and 0.2% respectively. Higher values of AP and JI indicate better performance, which is in contrast to the MR^−2^.

### 4.6. Comparison of Previous Works

We compare our method with some previous methods on the CrowdHuman and CityPersons datasets. We use three metrics to evaluate our method, AP, MR^−2^ and JI. AP is the abbreviation of average precision. AP represents both the precision and recall ratios of the detection results. A larger AP means better performance. MR^−2^, generally used in pedestrian detection, is short for log-average miss rate on false positive per image (FPPI) in [10^−2^, 100]. MR^−2^ is sensitive to false positives (FPs). A smaller MR^−2^ indicates better performance. JI evaluates how much the predicted boxes overlap with ground truths. A larger JI represents better performance.

The comparison results are listed in Table 3. For the sake of fairness, the methods listed above except ours are based on FPN. We set the IoU threshold of NMS as 0.5 for post-processing. As shown in Table 3, our method still obtains the best performance in all criteria, with 90.96% AP, 40.24% MR^−2^ and 83.12%. Compared with Cascade R-CNN, our method obtain 5.36% AP, 2.76% MR^−2^ and 2.52% JI gains on the validation set of CrowdHuman. We achieve higher improvements, 6.26% AP and 9.46% MR^−2^ compared with adaptive NMS. On the CityPersons dataset, our method also performs better than other methods. Although our method improves little, it indicates the good robustness of our method. As shown in Table 3, higher AP and JI mean that our method can detect more pedestrians correctly. Meanwhile, the improved MR^−2^ suggests that our method cannot introduce more false positives compared with other methods.

**Figure 5:**
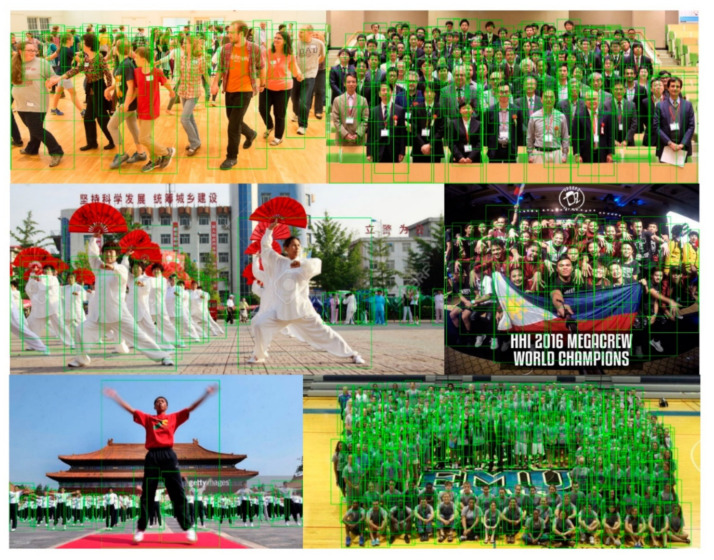
Detection results with our method. Although there are heavy occlusions in these images, the detector with our method can precisely detect the pedestrians.

**Figure 6:**
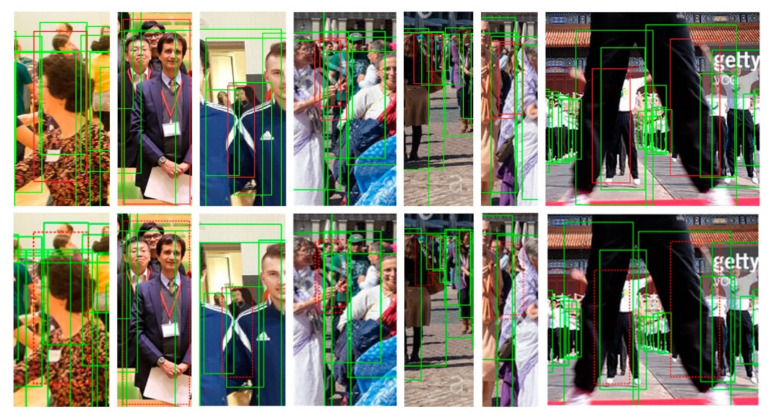
Comparisons of detection results between the ResNet50 and FPN baseline and our method. The images on the first row are the detection results by our method. The last row is the results by the baseline. The solid red boxes are the pedestrians detected by our method but not detected by the baseline method. In contrast, the dashed red boxes are the missed detections.

Figure 5 displays some detection results by our method. We can see that pedestrians in images can be detected correctly even though there is a heavy occlusion problem. Furthermore, we show the comparisons of the detection results between the ResNet50 and FPN baseline and our method in Figure 6.

We have boxed up the heavily occluded pedestrians correctly detected by our method with red solid boxes but not detected by the baseline with red dashed in contrast. That is, the dashed boxes are missed detections. Consequently, our method can deal with the heavy occlusion problem in pedestrian detection.

## 5. Conclusions

In this paper, we propose an effective network called double FPN based on ResNet, (abbreviated to DFR network), which can effectively extract and fuse features of images. It can not only extract rich and semantic information but also keep a complete contour, which improves the performance of the network towards heavily occluded targets. Besides, we put forward a new kind of loss function named repulsion loss of minimum, which can solve the occlusion from another perspective. Combining these two ideas, our network has achieved good performance on the CrowdHuman and CityPersons datasets.

## Figures and Tables

**Table 1:** Ablation study of feature maps. There are five different combinations.

Heatmaps	ResNet50	FPN	BiFPN	Double FPN	Separable Convolution(SC)	General Convolution(GC)
(**a**)	✓	✓				✓
(**b**)	✓		✓		✓	
(**c**)	✓		✓			✓
(**d**)	✓			✓	✓	
(**e**)	✓			✓		✓

**Table 2:** Ablation study implemented on the validation set of *CrowdHuman.*

Method	AP/%	MR^−2^/%	JI/%
ResNet50 + FPN baseline (impl. by [41])	85.8	42.9	79.8
ResNet50 + FPN (impl. by [41])	90.3	42.2	82.0
ResNet50 + BiFPN (Separable)	89.81	42.81	81.75
ResNet50 + BiFPN	90.64	40.82	82.83
ResNet50 + DoubleFPN (Separable w/o RLM)	90.76	41.05	82.83
ResNet50 + DoubleFPN (w/o RLM)	90.88	40.52	82.92
**ResNet50 + DoubleFPN (with RLM)**	**90.96**	**40.24**	**83.12**

**Table 3:** Comparisons with previous methods on the CrowdHuman and CityPersons datasets.

Dataset	Method	AP/%	MR^−2^/%	JI/%
CrowdHuman	FPN baseline (impl. by [41])	85.8	42.9	79.8
	ResNet50 + FPN (impl. by [41])	90.3	42.2	82.0
	FPN + Soft-NMS [28]	88.2	42.9	79.8
	Adaptive NMS [30]	84.7	49.7	—
	Cascade R-CNN [27] (impl. by [41])	85.6	43.0	80.6
	**Ours**	**90.96**	**40.24**	**83.12**
CityPersons	FPN baseline [41]	95.2	11.7	—
	FPN + Soft-NMS [28]	95.3	11.8	—
	ResNet50 + FPN [41]	96.1	10.7	—
	**Ours**	**96.23**	**10.64**	—

## Data Availability

The datasets involved in this paper are all public datasets. CrowdHuman: https://www.crowdhuman.org/download.html (accessed on 5 March 2021); CityPersons: https://www.cityscapes-dataset.com/ (accessed on 5 March 2021).
